# Extended embryo culture is effective for patients of an advanced maternal age

**DOI:** 10.1038/s41598-021-92902-9

**Published:** 2021-06-29

**Authors:** R. Sainte-Rose, C. Petit, L. Dijols, C. Frapsauce, F. Guerif

**Affiliations:** 1 Service de Médecine et Biologie de la Reproduction, CHRU de Tours, 2 bd Tonnelle, 37000 Tours, France; 2 Université François Rabelais de Tours, 37000 Tours, France; 3 UMR85 PRC, INRAE, 37380 Nouzilly, France; 4 UMR6175 PRC, CNRS, 37380 Nouzilly, France

**Keywords:** Developmental biology, Health care, Health occupations

## Abstract

The aim of this study was to determine the effectiveness of extended embryo culture in advanced maternal age (AMA) patients (37–43 years). In this retrospective analysis, 21,301 normally fertilized zygotes from 4952 couples were cultured until the blastocyst stage. Blastocyst development, including kinetics and morphology, transfer rate, implantation and live birth rates, were measured. In AMA patients, the blastocyst rate was significantly decreased as compared to that in younger women. On day 5, blastocysts underwent growth retardation in AMA patients, which was highlighted by a decreased rate of full/expanded blastocysts. Organization of the cells (trophectoderm and inner cell mass) was unaffected by age. However, in AMA patients, a ‘good’ morphology blastocyst had a decreased probability to implant compared with an ‘average’ morphology blastocyst in younger women. While the rates of blastocyst transfer and useful blastocysts were similar to younger patients, in AMA patients, both implantation and live birth rates were significantly reduced. Our results support the idea that extended embryo culture is not harmful for AMA patients. However, embryo selection allowed by such culture is not powerful enough to avoid chromosomal abnormalities in the developed blastocysts and therefore cannot compensate for the effect of a woman’s age.

## Introduction

Socials trends around the world have led women to delay becoming parents into their 30 s and, in some cases, their 40 s. In France, the average age of women giving birth has increased from 28.8 to 30.8 years over the last 20 years. Thus, this well-known physiological phenomenon is the most critical parameter determining the likelihood of conception, either naturally or following in vitro fertilization (IVF)^1,2^. Ageing of women is accompanied by reduced ovarian function^3^, lower oocyte quality^4^ and an increased rate of aneuploid oocytes^5^, leading to an extended rate of miscarriages^6^ and finally to a poor rate of live births^7^.

Embryo quality has always been considered an important predictor of successful implantation^8^. Previous studies have highlighted that extended embryo culture until the blastocyst stage is the best way to select useful embryos for transfer or cryopreservation^9,10^, with higher likelihoods of implantation in comparison with early cleaved embryos^11^. Considering that not all cleavage stage embryos will be able to reach the blastocyst stage, some patients may have no blastocysts for transfer^12,13^. Thereafter, blastocyst outcome (transfer, freezing and discarding) is based on morphological parameters. The most widely used blastocyst scoring system is arguably the one proposed by Gardner and Schoolcraft^14^, which takes into account three parameters (blastocyst stage expansion, inner cell mass and trophectoderm organization). Not surprisingly, higher implantation rates are observed with top-graded blastocysts^15,16^.

Many advanced maternal age (AMA) patients under care in IVF obviously have the hope to obtain embryos with good implantation potential. However, in such patients, the rate of aneuploidy at the blastocyst stage ranges from 30 to 85%, depending on the number of chromosomes studied^17–19^. While, chromosomal abnormalities can still be only identified by preimplantation genetic testing for aneuploidies (PGT-A), such promising technology is not used routinely by all IVF centres around the world^20^. As it has been shown that the extent of aneuploidy is lower in blastocysts in comparison with early embryos^21^, it could be expected that extended embryo culture could be useful to select embryos in AMA patients. However, due to a poorer response to ovarian stimulation, such patients have an increased probability to have less early embryos available for extended embryo culture and finally have a higher risk of transfer cancellation^22,23^.

The aim of this study was to evaluate the effectiveness of extended embryo culture in AMA patients. The points of measurement were the following: (i) biological outcome, including rate of blastocyst development, kinetics and morphology of blastocysts developed and rate of useful blastocysts; (ii) clinical outcome, including rate of transfer, clinical, live birth and implantation rates; and (iii) LBR (live birth rate) when blastocyst morphology and a woman’s age are combined.

## Results

### Biological outcomes

#### Overall blastocyst rate

**Figure 1:**
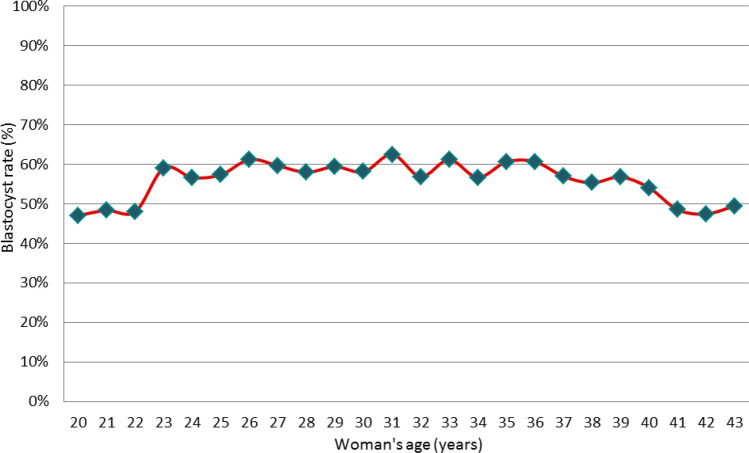
Blastocyst rate by the woman’s age. The blastocyst rate is the ratio between the number of blastocysts obtained at D5 or D6 and the number of normally fertilized zygotes at D1.

Not surprisingly, we found that the woman’s age had an impact on the overall blastocyst rate. Women aged 23–36 years had the highest average blastulation rate (59%, range 55–61%) (Fig. 1). Compared with the previous women, the blastocyst rate was significantly decreased for the two ‘extreme’ age groups: 48% in women aged 20–22 years (*P* < 0.05) and 54% in women aged 37–43 years (*P* < 0.05), with a significant decrease (48%) in women aged 41–43 years (*P* < 0.05). Due to the small number of young women available (n = 35), women aged 20–22 years were excluded from further analysis. In such small group the etiologies were the following: male (55%), combined (24%), female (21%, mainly polycystic ovary syndrome). Further studies including a higher number of young women would be mandatory to confirm such observation. General characteristics and biological outcomes in the four groups included in the study are described in Table 1.

**Table 1:** General characteristics and biological outcomes in the age groups of women.

	23–29 years	30–33 years	34–36 years	37–43 years	*p global*
No. of patients	1076	1522	1138	1216	
Woman’s age (years)	27.2 ± 1.7	31.6 ± 1.2	34.9 ± 0.8	39.0 ± 1.7	< 0.05
IVF technique	FIV = 35%	FIV = 40%	FIV = 42%	FIV = 39%	< 0.05
	ICSI = 65%	ICSI = 60%	ICSI = 58%	ICSI = 61%	
No. of total oocytes	10.8 ± 5.7	10.0 ± 5.2	9.6 ± 5.0	8.8 ± 4.4	< 0.05
No. of 2 PN zygotes	5.5 ± 3.4	5.1 ± 3.2^a^	5.0 ± 3.1^a^	4.5 ± 2.8	< 0.05
No. of TQE	1.2 ± 1.7	1.1 ± 1.5^a,b^	1.1 ± 1.5^a,c^	1.1 ± 1.5^b,c^	< 0.05
No. of blastocysts	3420	4499	3319	2919	
Blastocyst rate (%)	59%^a,b^	60%^a,c^	59%^b,c^	54%	< 0.05
Useful blastocyst rate (%)	68%	69%	68%	71%	NS
Freezing rate (%)	54%^a^	51%^a,b,c^	49%^b^	36%^c^	< 0.05

Numbers are expressed as mean ± standard deviation.

Values with the same superscript letters are not significantly different (*p* > 0.05).

*No.* number, *NS* non-significant, *PN* pronucleus, *TQE* top-quality embryo.

#### Kinetics of blastocyst development

**Figure 2:**
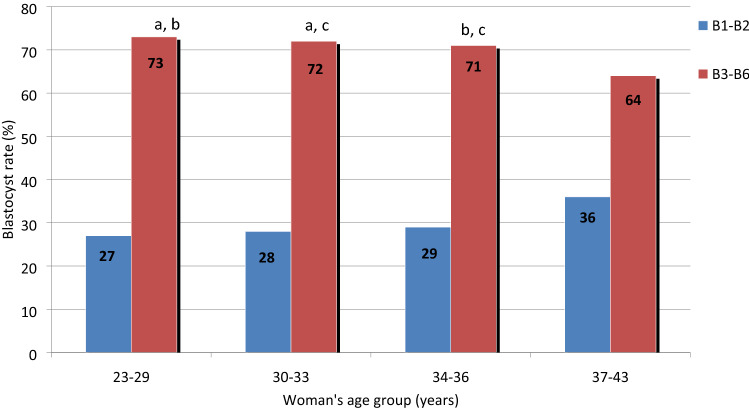
Stage of blastocyst expansion obtained at D5 as a function of the woman's age group. The blastocyst rate is the ratio between the number of blastocysts obtained at D5 or D6 and the number of normally fertilized zygotes at D1. B1: early blastocyst (the blastocoel being less than half the volume of the embryo). B2: early blastocyst (the blastocoel being greater than half the volume of the embryo). B3: full blastocyst (the blastocoel completely filling the embryo). B4: expanded blastocyst (the blastocoel volume now being larger than that of the early embryo and the zona starting to thin). B5: hatching blastocyst (the trophectoderm starting to herniate though the zona). B6: hatched blastocyst (the blastocyst having completely escaped from the zona) (from Gardner, D. K. & Schoolcraft, W. B, 1999)^25^. Columns with the same superscript letters are not significantly different (*P* > 0.05).

Among blastocysts developed after extended embryo culture, the proportion obtained at D5 was close to 70%, regardless of the woman’s age (range 71–73%). However, at D5, we observed that the stage of blastocyst expansion was slowed down in AMA patients (Fig. 2). Developed blastocysts from AMA patients have some growth retardation at D5 (more early blastocysts and fewer full/expanded/hatched blastocysts).

#### Cell organization of D5 blastocysts

**Figure 3:**
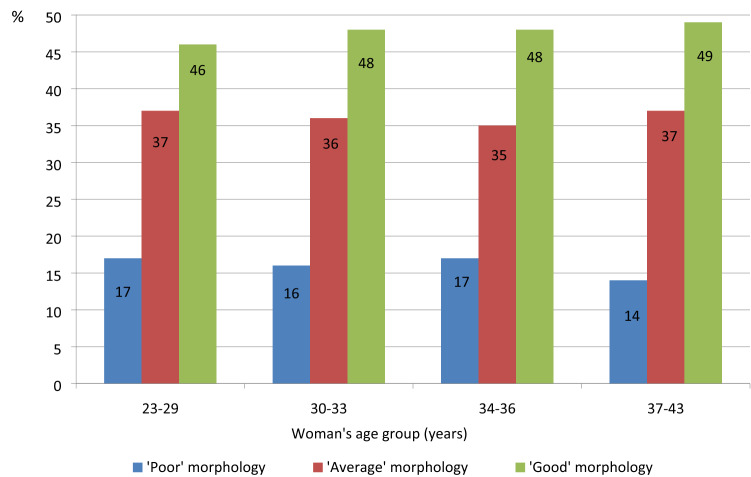
Morphology of D5 blastocysts according to the woman’s age group. Morphological assessment based on the expansion of the blastocoel cavity (B1–B6) and the number and cohesiveness of the inner cell mass (ICM) and trophectoderm (TE) cells^25^. For blastocysts graded as B3–B6, it is possible to assign independent scores to the ICM and TE. For the ICM, grade A indicated a tightly packed ICM with many cells; grade B, a loosely grouped ICM with many cells; and grade C, an ICM with very few cells. For the TE, grade A indicated a TE with many cells forming a cohesive epithelium; grade B, a TE with few cells forming a loose epithelium; and grade C, a TE with very few cells. ‘Poor’morphology: B3–B6, CC. ‘Average’ morphology: B3–B6, AC/CA/BC/CB). ‘Good’morphology (B3–B6, AA/AB/BA/BB). Data were not significantly different (*p* > 0.05).

There was no relationship between the woman’s age and organization of the TE, as well as the ICM (Fig. 3). The rate of ‘good’ morphology blastocysts was similar (46–49%) in AMA patients compared with other groups, showing that blastocyst morphology was not altered by the woman’s age.

#### Rate of useful blastocysts

The rate of useful blastocysts was similar between AMA patients and younger women, ranging from 68 to 71% (Table 1). When available, in AMA patients, as soon as the first IVF attempt, two blastocysts were transferred, whereas only one blastocyst was transferred in younger women for the first two IVF cycles. In AMA patients, fewer blastocysts were available for freezing (Table 1). Thus, the rate of cryopreservation was significantly lower in such patients compared with other groups: 36% versus 49–54%, respectively (*P* < 0.05).

### Clinical outcomes

AMA patients had the same probability (83%) to have a blastocyst transferred as younger patients, whereas patients aged 23–29 years had the highest probability (86%) (Table 2). Keeping in mind that the mean number of transferred blastocysts was significantly higher in AMA patients, in such patients, the clinical pregnancy rate as well as the LBR per oocyte retrieval were significantly decreased (Table 2). Not surprisingly, this result was at least partly explained by a higher miscarriage rate as age increases. This rate was l4% in women aged 23–36 years, while it reached 23% in AMA patients (Table 2). The rate of multiple pregnancies was not significantly influenced by the woman’s age, ranging from 8 to 13%. Overall, in AMA patients, the implantation rate was significantly lower compared with other groups: 30% versus 39–48%, respectively (*P* < 0.05).

**Table 2:** Clinical outcomes in the age groups of women.

	23–29 years	30–33 years	34–36 years	37–43 years	*p global*
Blastocyst transfer rate	86%^a,b^	82%^c,d^	83%^a,c,e^	83%^b,d,e^	< 0.05
No. of blastocysts transferred	1.1 ± 0.3^a^	1.1 ± 0.4^a,b^	1.2 ± 0.4^b^	1.5 ± 0.5	< 0.05
Implantation rate	47%^a^	48%^a^	39%	30%	< 0.05
Clinical pregnancy rate per oocyte pick-up	40%^a^	40%^a^	34%	30%	< 0.05
Miscarriage rate per clinical pregnancy	14%^a,b^	14%^a,c^	14%^b,c^	23%	< 0.05
Live birth rate per oocyte pick-up	33.5%^a^	33%^a^	29%	22%	< 0.05
Multiple pregnancy rate per live birth	8%	8%	10%	13%	NS

Values with the same superscript letters are not significantly different (*p* > 0.05).

*No* number, *NS* non-significant.

### LBR by transfer according to D5 blastocyst morphology and woman’s age

**Figure 4:**
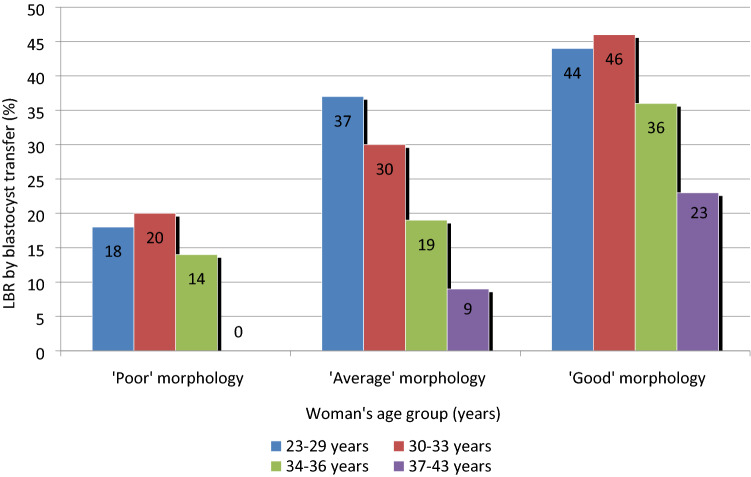
Live birth rate (LBR) by transfer according to D5 blastocyst morphology and the woman’s age group. LBR: live birth rate. Morphological assessment based on the expansion of the blastocoel cavity (B1–B6) and the number and cohesiveness of the inner cell mass (ICM) and trophectoderm (TE) cells^25^. For blastocysts graded as B3–B6, it is possible to assign independent scores to the ICM and TE. For the ICM, grade A indicated a tightly packed ICM with many cells; grade B, a loosely grouped ICM with many cells; and grade C, an ICM with very few cells. For the TE, grade A indicated a TE with many cells forming a cohesive epithelium; grade B, a TE with few cells forming a loose epithelium; and grade C, a TE with very few cells. ‘Poor’morphology: B3–B6, CC. ‘Average’ morphology: B3–B6, AC/CA/BC/CB). ‘Good’morphology (B3–B6, AA/AB/BA/BB).

The LBR by transfer according to D5 blastocyst morphology depends on the woman’s age (Fig. 4).When considering full/expanded/hatched blastocysts, we observed first that, in all age groups, the LBR per transfer increased with improvement of blastocyst morphology. For example, in patients aged 30–33 years, the LBR increased gradually from 20% (‘poor’ morphology) to 30% (‘average’ morphology), then reached 46% (‘good’ morphology) (Fig. 4). A similar trend was observed for AMA patients, despite lower values for each morphological stage (Fig. 4).

Second, when a given morphological stage was considered (‘poor’, ‘average’ or ‘good’), the LBR tended to decrease with age. Thus, the transfer of blastocysts with ‘good’ morphology was associated with a LBR per transfer of 23% in women aged 37–43 years, while it reached 44–46% in women aged 23–33 years. Similarly, the transfer of blastocysts with ‘average’ morphology was associated with a LBR per transfer of 9% in women aged 37–43 years, while it reached 30–37% in women aged 23–33 years. Finally, the transfer of blastocysts with ‘poor’ morphology was associated with a LBR per transfer of 0% in women aged 37–43 years, while it reached 18–20% in women aged 23–33 years. In AMA patients, a ‘good’ morphology blastocyst is less probable to implant compared with an ‘average’ morphology blastocyst in women aged 23–33 years, 23% versus 34%, respectively (*P* < 0.05).

## Discussion

Previous studies have reported improved results after the transfer of fresh blastocysts. As a result, extended embryo culture until the blastocyst stage is now performed much more often^26^. Moreover, with the efficacy of vitrification, frozen blastocysts are able to provide similar (or even better) implantation rates compared to those obtained after fresh transfers^27,28^. However, one may question whether such good results are available for all couples. This topic is particularly relevant for AMA patients, keeping in mind that the threshold to define such women remains highly variable^7,29,30^.

In a previous study including 289 IVF cycles, the authors showed that, after co-culture, preimplantation development to blastocysts declined in patients above 30 years of age due to an increase in embryo arrest^29^. Similarly, Pantos observed that women aged 40 years and over had a decreased probability to have at least a blastocyst (after culture with sequential media) than women under 40 years of age (22% vs. 41%, respectively, n = 293)^30^. Another study analysing 397 IVF cycles showed a significantly lower blastocyst rate in women over 39 years of age (29% vs. 55%) after culture with sequential media^7^. In women aged 42 years and older, fewer embryos were able to reach the 8-cell stage compared to women under 38 years of age (66% vs. 72%, respectively); a higher percentage of embryos stopped at 4–7 cells^31^. It is well established that the activation of the embryo’s genome begins at the 4–8 cell stage^32^. The reduced blastocyst rate observed in AMA patients could be partly explained by a lower ability of their early embryos to go through this activation. In contrast to previous studies, strengths of our study are the number of women included (n = 4952) and the large number of normally fertilized zygotes (n = 21,301) cultured until the blastocyst stage. In accordance with the previous studies described above, the blastocyst rate decreased slightly at 37 years of age, then more drastically at 41 years of age. This observation could be at least partly explained by the fact that the rate of aneuploidy at the blastocyst stage was particularly high in AMA patients, ranging from 30 to 85%, depending on the number of chromosomes studied^17–19^. Such a hypothesis is bolstered by data from blastocyst biopsies at D5 for comprehensive chromosome analysis^19^. The lowest rate of aneuploid embryos (20–27%) was found in women between 26 and 30 years of age. Then, this rate increased gradually to reach 85% in women aged 43 years and older.

According to our data, when the kinetics of blastocyst development is evaluated according to the day of blastocyst development (D5 vs. D6), AMA patients have similar results compared with other groups. A recent study including 785 patients showed that, among a selection of euploid blastocysts, the proportion of blastocysts obtained at D5 was the same regardless of the woman’s age^2^. More accurately, we also observed that the developed blastocysts from AMA patients had some growth retardation at D5 (fewer full/expanded/hatched blastocysts and more early blastocysts). Such results are in agreement with two other studies showing that, with age, women were less likely to have an expanded blastocyst^29,33^. Moreover, it has been shown that the odds ratio of having a better blastocyst expansion score at day 5 was lower for embryos with single chromosomal aneuploidy than euploid embryos^34^.The fact that a higher rate of aneuploidy is observed among early stage blastocysts compared with higher expansion grade blastocysts could support these data^35,36^. By contrast, such observations cannot be explained by already published data from time lapse studies focusing only on early morphokinetic variables. Those authors demonstrated that age had no effect on the kinetic parameters of the first 3 days of embryo development^31,37^. To our knowledge, only one study has reported altered kinetic variables (tPNf, t2, t3 and t4) for embryos produced by women aged 30–40 years compared with younger women aged 20–30 years^38^.

According to our findings, at D5, the organization of cells from the TE and ICM was unaffected by woman’s age, with a similar rate of ‘good’ morphology blastocysts between AMA patients and other groups. In agreement with our observation, Irani et al. showed that cell organization in 870 euploid blastocysts was not affected by the woman’s age^2^. Indeed, the proportion of ‘good’ morphology blastocysts was 16% in women under 35 years old and 14% in those over 42 years old. Interestingly, in AMA patients, we observed that a ‘good’ morphology blastocyst will have a decreased probability to implant compared with an ‘average’ morphology blastocyst in women aged 23–36 years. This finding might be related to substantial genetic abnormalities, including monosomy and complex aneuploidy, in apparently normal blastocysts^39^. It has already been shown that assessing blastocyst morphology cannot reliably predict ploidy status^35,39,40^. Some aneuploidy embryos are capable of achieving ‘good’ morphology, whereas some euploid embryos are of ‘poor’ morphology. Consequently, morphologic analysis cannot be relied on to ensure transfer of chromosomally normal embryos. Thus, it seems that the rate of aneuploidy increases with age, without altering the organization of the cells. The management of blastocyst PGT-A cycles suggests that the commonly used parameters of blastocyst evaluation are not good enough indicators to improve the selection of euploid embryos^41^.

A well-known drawback with extended embryo culture is the lack of blastocysts for transfer due to embryo culture arrest. Thus, one may be afraid to delay embryo transfer until the blastocyst stage for couples with a poor prognosis, such AMA patients. However, in our study, the overall blastocyst transfer rate was relatively stable and high (90%) for women between 23 and 43 years of age. Our results disagree with some older articles showing that the transfer rate decreased significantly^29^ or tended to decrease^30^ when the maternal age increased. Such a discrepancy may be explained by the evolution of extended culture over the last 20 years, with an improvement in culture conditions with time. Thus, nowadays, increased maternal age should not hinder extended embryo culture in IVF protocols.

Unsurprisingly, our results showed that both the implantation rate and LBR are altered by the woman’s age, with poor results for AMA patients. These results are in agreement with other studies showing higher miscarriage rates and lower live birth rates in patients over 39 years of age^7,17,30^. Such observations may be explained by the fact that aneuploidy is more frequent in AMA patients^42^. Reporting IVF outcomes using cumulative LBR following the use of all fresh and frozen embryos derived from a single IVF cycle appears to be a better measure of IVF treatment success. In our study, the rate of useful (transferred or cryopreserved) blastocysts was similar between AMA patients and women from other groups. However, in AMA patients, as more blastocysts are used from fresh transfers, fewer blastocysts are available for freezing. As a consequence, compared with young women, AMA patients are exposed to poor cumulative LBR^43^.

Interestingly, two recent studies compared two embryo culture strategies (extended embryo culture or embryo transfer on Day 3) in AMA patients^13,44^. In a first study, the purpose was to determine whether all-blastocyst-culture can benefit AMA patients with low ovarian reserve^13^. Multivariate analysis showed that female age and the number of D3 transferrable embryo are risk factors for the failure of blastocyst culture. The authors drew to conclusion that all-blastocyst-culture will not adversely affect the pregnancy outcome of AMA patients with low ovarian reserve. In the second one, in older women (aged > 38 years) with four or fewer D3 embryos, cumulative LBR were similar between the two groups^44^. In agreement with our study, the authors argued that blastocyst culture and transfer could significantly increase pregnancy rate per embryo transfer cycle and might be appropriate in some older patients with a good clinical prognosis. In conclusion, for numerous reasons, more and more women are facing age-related infertility problems requiring IVF assistance. In the same way, extended embryo culture has been widely developed in recent years, with promising results in terms of implantation rates in comparison with early embryo transfers. Our data argue that there is no evidence to exclude AMA patients from extended embryo culture. However, the couple’s information needs to highlight the chances of success as a function of the woman’s age. In AMA patients, the poorer results seem independent of extended culture but are explained mainly by chromosomal abnormalities. Putting aside the particular rescue of PGT-A in AMA patients, such couples should understand that IVF cannot counterbalance the inexorable effect of the biological clock. However, our results highlight that blastocyst embryo culture is not harmful in AMA patients. It could be as useful and effective in such patients in comparison with younger patients, and good chances of reaching a blastocyst transfer are contemplated in AMA patients.

## Methods

### Study design

A retrospective study was undertaken in the IVF unit, Bretonneau University Hospital, Tours, France, between January 2011 and December 2019. Over these 9 years, we have collected all IVF attempts (including conventional IVF and intracytoplasmic sperm injection (ICSI)) while excluding oocyte donation and ICSI with testicular extractions. All methods were carried out in accordance with relevant guidelines and regulations. Our experimental protocol was approved by ERERC (Espace de Réflexion Ethique de la Région Centre) which is the Ethics Committee for research involving human subjects in our hospital. Informed consent was obtained from all subjects. Data were collected from 4952 patients, representing 21,301 normally fertilized zygotes and 14,157 blastocysts developed at day 5 (D5) or day 6 (D6). In our study, the average age of the women was 33.1 ± 4.5 years.

Our focus was the impact of the woman’s age on extended embryo culture. The blastocyst rate was calculated for each individual age data, without preconceptions (Fig. 1). Following this analysis, we grouped the women into four age groups with similar sizes: 23–29 years, 30–33 years, 34–36 years and 37–43 years. Due to the low number (n = 35) of women under the age of 23 available in our study, such patients have been excluded for further detailed evaluation. Women 37–43 years of age were referred as AMA patients. The distribution of the AMA over the time period (2011–2019) studied was even, ranging from 22 to 27%.

### IVF procedure

The ovarian stimulation protocol and the IVF and ICSI procedures used have already been described elsewhere^24^. Briefly, from 2011 to 2019, embryo culture with sequential medium and assessment were carried out as follows: fertilization (day 0) was performed in IVF Fertilization Medium (VITROLIFE, Göteborg, Sweden). The following morning (day 1), the oocytes were individually placed in microdrops (25 µl) in IVF G1 Medium (VITROLIFE) under Ovoil (VITROLIFE). From day 3 to 5/6, embryo culture was performed in microdrops in IVF G2 Medium (VITROLIFE) under mineral oil. All cultures were grown in MINC benchtop incubators (K-MINC, COOK MEDICAL, Brisbane, Australia) at 37 °C with 6% CO_2_, 5% O_2_ and 89% N_2_.

### Morphological assessment of early embryos

All the subsequent optical assessments were performed by two experienced embryologists, using an inverted microscope with Hoffman modulation contrast (200 × and 400 × magnification). Embryos were evaluated 44–46 h post-insemination/ICSI (day 2) on the basis of the number of blastomeres, shape (regularity) of cells, fragmentation rate and presence of multinucleated blastomeres. At this early stage, embryos with multinucleated blastomeres were excluded from further extended embryo culture. According to ASEBIR consensus scoring, a top-quality embryo (TQE) on day 2 was defined as follows: four even blastomeres with less than 10% fragmentation and no vacuoles or multinucleation^10^.

### Biological outcome

The blastocyst rate is the ratio between the number of blastocysts obtained at D5 or D6 and the number of normally fertilized zygotes at D1. At D5 or D6, a blastocyst is considered ‘useful’ when it is used for transfer or freezing.

### Assessment of blastocyst morphology

The outcome of extended embryo culture was recorded for each individually cultured embryo. The morphological assessment was based on the expansion of the blastocoel cavity (B1–B6) and the number and cohesiveness of the inner cell mass (ICM) and trophectoderm (TE) cells^25^. When an embryo had started to expand (for blastocysts graded as B3–B6), it was then possible to assign independent scores to the ICM and TE. This next step of the grading was designed to be performed on an inverted microscope. For the ICM, grade A indicated a tightly packed ICM with many cells; grade B, a loosely grouped ICM with many cells; and grade C, an ICM with very few cells. For the TE, grade A indicated a TE with many cells forming a cohesive epithelium; grade B, a TE with few cells forming a loose epithelium; and grade C, a TE with very few cells.

We categorized four morphological groups according to the stage of expansion and organization of the cells: ‘early’ (B1–B2), ‘good’ (B3–B6, AA/AB/BA/BB), ‘average’ (B3–B6, AC/CA/BC/CB) and ‘poor’ (B3–B6, CC). Only supernumerary blastocysts with the following characteristics were frozen on day 5/6: B3–B6 stages with morphology classified as ‘good’ or ‘average’.

### Transfer strategy

The transfer strategy in our IVF centre takes into account two main criteria: the woman’s age and the rank of the oocyte retrieval. The decision to schedule a transfer at the cleavage-stage embryo on day 2 or at the blastocyst stage was taken by the physician and the couple together. Couples were clearly informed by a physician regarding the risks of multiple pregnancies following the transfer of two blastocysts. Moreover, theoretical drawbacks (uncertainty of reaching the blastocyst stage) and advantages (embryo selection after genome activation, more accurate synchrony between blastocyst and endometrium, and lower uterine contraction at the time of blastocyst transfer) of extended culture were explained. A single blastocyst transfer is recommended when the woman is under the age of 37 years old and undergoing a first or a second IVF attempt. A transfer of two blastocysts is recommended for all women from the third oocyte retrieval and as soon as the first IVF attempt from the age of 37 and onwards.

### Clinical outcome

The serum human chorionic gonadotropin (hCG) level was measured 7 days after blastocyst transfer. Clinical pregnancy was defined as the presence of a gestational sac with foetal heart activity on ultrasound examination 5 weeks after oocyte retrieval. The implantation rate was defined as the number of gestational sacs divided by the number of blastocysts transferred. The live birth rate by oocyte retrieval was the final clinical outcome.

### Statistical analysis

Statistical analysis was performed using Statview 5.1 software (ABACUS Concepts, Berkeley, CA, USA). Quantitative variables were compared using variance analysis, followed by Fisher's least significant difference (LSD) test. Qualitative data were compared using contingency tables (chi-squared test). Differences were considered significant with a *P* < 0.05.
